# Integrated Chinese Herbal Medicine and Western Medicine on the Survival in Patients with Colorectal Cancer: A Retrospective Study of Medical Records

**DOI:** 10.1155/2020/4561040

**Published:** 2020-01-13

**Authors:** Ming-Hsien Yeh, Hung-Pin Chiu, Mei-Chun Wu, Malcolm Koo, Nai-Wei Lin, Kou-Kai Liao, Chia-Chou Yeh, Te-Mao Li

**Affiliations:** ^1^Graduate Institute of Chinese Medicine, China Medical University, Taichung 40402, Taiwan; ^2^Department of Chinese Medicine, Dalin Tzu Chi Hospital, Buddhist Tzu Chi Medical Foundation, Dalin, Chiayi 62247, Taiwan; ^3^Department of Information Management, Nanhua University, Dalin, Chiayi 62249, Taiwan; ^4^Graduate Institute of Long-term Care, Tzu Chi University of Science and Technology, Hualien 97005, Taiwan; ^5^Dalla Lana School of Public Health, University of Toronto, Toronto, Ontario M5T 3M7, Canada; ^6^Institute of Computer Science and Information Engineering, National Chung Cheng University, Minxiong, Chiayi 621, Taiwan; ^7^School of Post-Baccalaureate Chinese Medicine, Tzu Chi University, Hualien 97004, Taiwan

## Abstract

Recent studies suggested that Traditional Chinese Medicine could play a beneficial role in conventional cancer treatment. The aim of this retrospective cohort study was to investigate the effect of Chinese herbal medicine (CHM) combined with Western medicine on the survival of patients with colorectal cancer. A retrospective cohort study was conducted on patients with newly diagnosed colorectal cancer identified from the Dalin Tzu Chi Hospital Cancer Registry Database in 2004–2014. Combining with the medical records of the study hospital, patients were classified into CHM users and CHM nonusers. Kaplan–Meier analyses and Cox proportional hazards regression analyses were used to investigate the survival between CHM users and CHM nonusers. A total of 535 patients with colorectal cancer were included in the study with 147 of them were CHM users. The log-rank test for Kaplan–Meier survival curve revealed a statistically significant difference between the survival of CHM users and CHM nonusers (P=0.006). Results from multivariate Cox regression analysis showed that CHM use was significantly associated with better survival (adjusted hazard ratio = 0.54, 95% CI = 0.38 to 0.77). In addition, the CHM formulae Jia Wei Xiao Yao San, Zhi Bah Di Huang Wan, Ping Wei San, and Qui Pi Tang were significantly associated with better survival. In conclusion, findings from this retrospective cohort study indicated that integrated CHM and Western medicine could improve survival in patients with colorectal cancer. Additional research on integrating TCM with Western medicine to improve cancer survival is warranted.

## 1. Introduction

Colorectal cancer is a leading cause of cancer death worldwide and is the third leading cause of cancer mortality in Taiwan [1]. While the overall five-year survival rate of colorectal cancer has exceeded 65% in most affluent countries [2], it is only 9–12% for patients with stage IV colorectal cancer [3, 4]. Currently, the mainstream treatment of colorectal cancer is laparoscopic surgical resection for primary disease, but radiotherapy and chemotherapy are often required for metastatic disease [5]. In addition to Western medical treatments, Traditional Chinese Medicine (TCM) has been used in the treatment of patients with cancer [6]. TCM has been reported to be able to alleviate chemotherapy nausea [7], reduce pain [8], improve the curative effect of cancer treatment, enhance quality of life, and reduce adverse events [9]. Research indicated that certain Chinese herbal medicines (CHM), such as *Salvia miltiorrhiza*, could inhibit the proliferation of colorectal cancer cells by apoptosis [10], thereby effectively prolonging survival [11–14].

A secondary analysis of the Taiwan's National Health Information Research Database (NHIRD), a nationwide, population-based medical claim records database, showed that Xiang Sha Liu Jun Zi Tang (7.1%), Bu Zhong Yi Qi Tang (4.3%), and Jia Wei Xiao Yao San (4.1%) were the top three most commonly prescribed single Chinese herbal medicine (CHM) formulae prescribed for postsurgery colon cancer patients [11]. However, the study did not analyze the effect of these formulae on survival rates. In addition, the NHIRD did not contain information on cancer stage, chemotherapy, radiotherapy, and surgery [15], and hence their potential confounding effects could not be controlled. Alternatively, the use of the Taiwan Cancer Registry (TCR) could overcome this data limitation [16]. The aim of this retrospective cohort study was to investigate the effect of CHM combined with Western medicine compared with Western medicine alone on the survival of patients with colorectal cancer, using local hospital medical record data linked with the TCR. In addition, the effectiveness of the 10 most commonly used CHM formulae with different dosages on survival was investigated.

## 2. Materials and Methods

### 2.1. Data Source

The data sources of this study comprised the TCR of Dalin Tzu Chi Hospital [16] and medical records database of the study hospital from 2004 to 2014. First, we used TCR to identify patients with newly diagnosed colorectal cancer. Their treatment, clinical stage, and cause of death were also obtained from the TCR. Only deaths due to colorectal cancer were considered as the mortality outcome in this study. Diagnosis codes for cancer types were based on the ICD for Oncology, 3rd edition (ICD-O-3), from 2002. The medical records of the study hospital were used to ascertain basic and clinical characteristics of the patients and also the type, dosage, frequency, and treatment duration of CHM used. Disease diagnoses were based on the International Classification of Diseases, Ninth Revision, Clinical Modification (ICD-9-CM). Study participants had to be treated for at least 30 days at Dalin Tzu Chi Hospital to be eligible for the study. The study protocol was approved by the institutional review board of Dalin Tzu Chi Hospital, Buddhist Tzu Chi Medical Foundation (IRB no. B10503013).

### 2.2. Study Design and Exposure Assessment

We conducted a retrospective cohort study to examine the association of CHM use and survival rates in patients with colorectal cancer. To avoid immortal time bias, patients were followed starting from 3 months after their first colorectal cancer diagnosis [17] to the date of death or the end of the study period, whichever came first. A total of 1209 patients with colorectal cancer (ICD-9-CM: 153–154.8 and ICD-O-3: C18.0–C21.8) between 2004 and 2014 were identified from the TCR. We excluded patients whose (1) survival status was unclear or death reason was unknown (code number: 7777 or 7798), or (2) cancer stage unknown or stage 0. A total of 535 eligible patients were included.

Study patients were classified as either CHM users or CHM nonusers based on whether their cumulative days of CHM use were more than 30 days during the first year following the first diagnosis date of their colorectal cancer. CHM use was considered as interrupted when the interval between two consecutive CHM treatments exceeded six weeks.

### 2.3. Outcome Assessment

The primary outcome of this study was colorectal cancer-specific survival. Survival time was calculated in months from the first date of colorectal cancer diagnosis to either death or the end of the study period. Potential confounding variables included age, sex, comorbidities at baseline, clinical stage of cancer, and treatment of colorectal cancer (surgery, radiotherapy, and chemotherapy). According to the National Health Insurance (NHI), the mortality rate of colorectal cancer increases considerably after the age of 45 years. Therefore, the patients were stratified into three subgroups by age (≤45 years, 46–65 years, and >65 years) in all the analyses.

Based on individual medical records one year before initial cohort entry, the Charlson–Deyo comorbidity index was used to assess the presence of comorbidity [18]. Following the classification system developed by the American Joint Committee on Cancer (AJCC 7th edition), clinical stage was assigned I, II, III, or IV. Uncertain information (code number: 888 or 999) was regarded as an unknown stage. Metastasis was recorded as yes or no.

For a specific CHM, the total dosage taken by each patient was calculated by multiplying days, doses, and frequencies of treatments. Based on the overall dose of medication, we obtained the top 10 commonly used CHM. For each of the 10 CHM formulae, patients taking dosage higher than the median were classified as the high-dose group, otherwise as the low-dose group.

### 2.4. Statistical Analysis

We used Chi-squared tests to compare the proportions of demographic variables, clinical stage of cancer, comorbidity variables, and treatment methods (surgery, chemotherapy, and radiotherapy) in the two groups. The Kaplan–Meier survival curve and log-rank test were used to compare survival curves. Univariate and multivariate Cox proportional hazards regression analyses were used to examine the hazard ratio (HR) of survival for CHM use and for dosage used. The multivariate-adjusted model was developed using backward elimination based on the likelihood ratio test. All *P* values less than 0.05 were considered statistically significant.

## 3. Results

### 3.1. Characteristics of Patients with Colorectal Cancer

The study identified 535 colorectal cancer patients; 388 were CHM nonusers, and 147 were CHM users.  Table 1 shows a comparison of age and sex distribution of the source population (1,209 patients) with our final study sample (535 patients). The distribution of sex and age group was not significantly different between the source population and our final sample.

Table 2 indicates that clinical stage, age, sex, treatment (surgery, radiotherapy, and chemotherapy), distant metastases, comorbidity score 2, and comorbidity score 6 did not differ significantly between CHM users and nonusers. However, the proportion of comorbidity score 1 in CHM users is considerably higher than that in CHM nonusers (*P*=0.010).

### 3.2. Survival by CHM Use

The mean follow-up period for all 535 patients was 36.0 months (SD = 22.8). The mean follow-up period for the CHM nonusers was 35.2 months (SD = 23.9), ranging between 3.0 and 90.9 months; the mean follow-up period for the CHM users was 38.1 months (SD = 19.7), ranging between 3.5 and 91.4 months. Overall, 197 deaths (36.8%) occurred during the study period. The CHM nonusers group had 156 deaths (40.2%), and the CHM users group had 41 deaths (27.9%).

**Figure 1:**
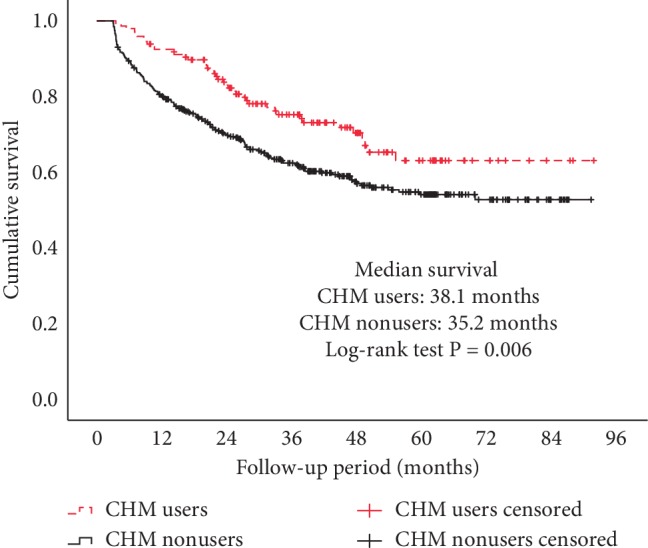
Kaplan–Meier survival curves for patients with colorectal cancer by Chinese herbal medicine (CHM) use.

The log-rank test for Kaplan–Meier survival curves revealed a statistically significant difference between the survival of the two groups (*P*=0.006) (Figure 1). The survival rates of the first, third, and fifth years for CHM nonusers were 80%, 62%, and 54%, while for CHM users were 92%, 75%, and 63%.

**Figure 2:**
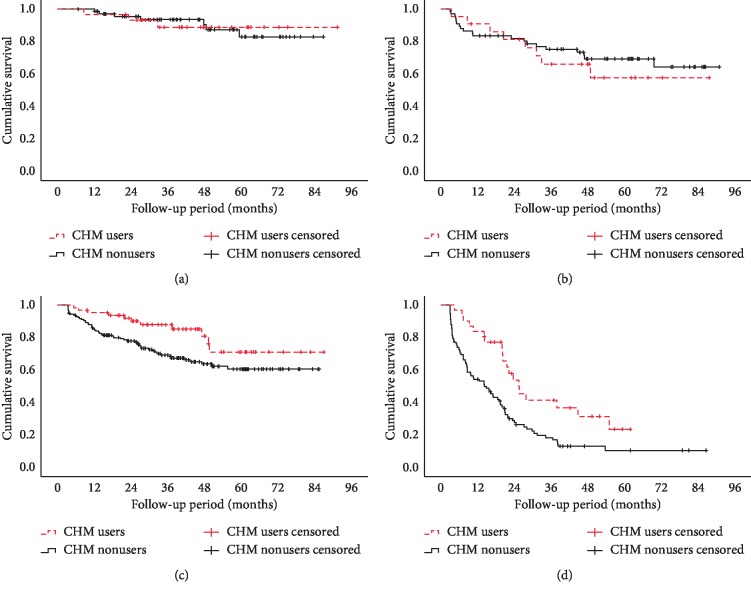
Kaplan–Meier survival curves for patients with colorectal cancer by Chinese herbal medicine use: (a) stage (I), (b) stage II, (c) stage III, and (d) stage IV.

Further analyses revealed that there were no significant differences in the survival curves of stages I (*P*=0.966) and II (*P*=0.581) cancer (Figures 2(a) and 2(b)), but there were significant differences in the survival curves of stages III (*P* = 0.027) and IV (*P* = 0.003) cancer (Figures 2(c) and 2(d)).

### 3.3. Cox Regression Analysis of Patients with Colorectal Cancer

The univariate Cox regression analysis showed a significant association between the use of CHM and increased survival (HR = 0.62, 95% CI = 0.44–0.87, *P*=0.007) (Table 3). After adjusting for potential confounders, the use of CHM remained significantly associated with better survival (adjusted HR = 0.54, 95% CI = 0.38 to 0.77, *P*=0.001). Results of the multivariate analysis also revealed that the cancer stage was significantly associated with survival (stage 2-adjusted HR = 2.98, 95% CI = 1.44 to 6.18, *P*=0.003; stage 3-adjusted HR = 4.28, 95% CI = 2.18 to 8.42, *P* < 0.001; stage 4-adjusted HR = 17.49; 95% CI = 8.90 to 34.38; *P* < 0.001). In addition, surgery and chemotherapy were associated with better survival with adjusted HRs of 0.29 (95% CI = 0.19 to 0.43; *P* < 0.001) and 0.47 (95% CI = 0.34 to 0.65; *P* < 0.001), respectively.

### 3.4. Top 10 Commonly Used CHM on Survival

Based on the total dosage of 147 CHM users, the top 10 used herb formulae are presented in Table 4. As shown in Table 5, certain Chinese herbal formulae were found to be associated with better survival. Univariate Cox hazards analyses showed that the use of high doses of Jia Wei Xiao Yao San (HR = 0.34, 95% CI = 0.14 to 0.82, *P*=0.017) and Liu Wei Di Huang Wan (HR = 0.12, 95% CI = 0.02 to 0.89, *P*=0.038) was significantly associated with better survival. After adjusting for potential confounding factors, the use of high doses of Jia Wei Xiao Yao San (adjusted HR = 0.38, 95% CI = 0.16 to 0.93, *P*=0.035), Ban Xia Xie Xin Tang (adjusted HR = 0.32, 95% CI = 0.10 to 1.00, *P*=0.049), Ping Wei San (adjusted HR = 0.31, 95% CI = 0.11 to 0.84, *P*=0.022), and Qui Pi Tang (adjusted HR = 0.26, 95% CI = 0.11 to 0.59, *P*=0.001) was significantly associated with better survival. In addition, the use of low doses of Jia Wei Xiao Yao San was significantly associated with better survival (adjusted HR = 0.40, 95% CI = 0.20 to 0.79, *P*=0.009).

Table 6 shows the daily dosage and duration of administration for the four aforementioned significant CHM. The daily dosage ranged from 2.92 g for Ping Wei San to 4.68 g for Qui Pi Tang. The duration ranged from 25 days to 63 days for low and high doses of Jia Wei Xiao Yao San, respectively.

## 4. Discussion

In this retrospective cohort study, significant survival benefits for patients with colorectal cancer receiving treatment of integrated Chinese and Western medicine compared with Western medicine alone was observed, particularly among patients with stage III and IV cancer. Results from multivariate analysis, adjusting for cancer stage, surgery, and chemotherapy, showed that the hazards significantly decreased by 46% in CHM users compared with nonusers. Previous research also showed that the use of CHM could prolong the survival time of patients with colorectal cancer [11, 19].

Among the top 10 commonly used CHM, high doses of Jia Wei Xiao Yao San, Zhi Bah Di Huang Wan, Ping Wei San, and Qui Pi Tang were associated with better survival. To induce the therapeutic effect, daily dosages ranged between 2.92 and 4.68 g, and treatment duration of 25 to 63 days was required. First, Jia Wei Xiao Yao San is generally the primary herb for treating cancers. It features emotional regulation, evacuation of Qi depression, and relief of gastrointestinal distress. The therapeutic effect of this herb could be through the alleviation of psychosomatic stress as a result of colon cancer treatment [20]. With reasonable doses, Jia Wei Xiao Yao San is safe and does not interact with 5-FU in conventional treatment [21].

Second, we found that Zhi Bah Di Huang Wan could significantly improve the survival patients with colorectal cancer. Patients with colon cancer and type II diabetes mellitus were shown to have a significantly shorter overall survival rate [22]. Zhi Bah Di Huang Wan could exhibit a synergistic effect of reducing blood glucose or increasing insulin sensitivity and delaying the insulin resistance of cells [23, 24].

Third, patients receiving sphincter-preserving surgery, especially when it is combined with radiotherapy often experience bothersome changes in bowel habits, such as fecal incontinence, frequent bowel movements, urgency, and emptying difficulties, which can lead to a low quality of life [25, 26]. Pin Wei San could reduce flatulence in the gastrointestinal tract for cancer patients suffering from side effects, such as nausea and vomiting [14].

Fourth, Qui Pi Tang can be used to treat amnesia, fatigue, poor memory or forgetfulness, anorexia, anemia, insomnia, palpitation, and neurosis [27]. It can also treat blood stools, subcutaneous purple spots, diet reduction, pain, and severe insomnia [28–30]. Moreover, Qui Pi Tang has been reported to exert antistress effects and beneficial effects on gastrointestinal and immune-mediated diseases [31], which are the common side effects of colorectal cancer after radiotherapy and chemotherapy.

There are a few limitations worth noting in this study. First, the research only chooses to confirm the diagnosis of Western medicine at a single regional hospital, as well as the patients who also receive the CHM treatment in outpatient and consultation. Alternative therapies are common in the United States (45.0–51.8%) [19, 32] and Norwegian (33.8%) [33]. There were 27.48% CHM users in this study; in contrast, only 12–20% of colon cancer patients in the NHIRD database receive CHM treatment [34]. However, our research findings are in line with prior studies, showing a higher survival rate and longer survival time for CHM users. Thus, the difference would be reasonably overlooked.

Second, the possibility of selection bias could not entirely be ruled out because 674 (55.7%) out of 1,209 patients identified from the TCR were excluded from the analysis due to missing information on survival status or cancer stage. Nevertheless, comparison of the age and sex distribution between the source population and the study sample did not reveal any significant differences.

Third, CHM use corresponds to TCM syndrome differentiation, yet the treatment by Chinese medicine practitioners is highly individualized. Practically, according to the syndrome types of patients, the prescriptions and the herbal formulae might differ. Syndrome differentiation is diagnosed by Chinese medicine practitioners, lacking a scientific system to provide more delicate cluster analysis. Consequently, it is urgent to apply artificial intelligence to develop automatic TCM syndrome differentiation system [35]. As such, “syndrome type” can be classified by the system, and more accurate CHM use can be obtained for TCM patients. In this case, we could expect to dig deeper into the effectiveness of CHM use.

Fourth, we focused on colorectal cancer-specific mortality as the outcome of this study. Cause of death due to complications, such as cardiac arrest, at the end-stage of colorectal cancer would not be included in the survival analysis. Nevertheless, differential misclassification, if present, in the cause of death due to complications should not be different between the CHM users and CHM nonusers groups. In addition, since the use of chemotherapy was marginally higher in the CHM users group compared with the CHM nonuser group (*P*=0.069), it might increase the mortality due to cardiac events in the CHM users group. However, the potential confounding effect of chemotherapy was adjusted in the final multivariate Cox model. Therefore, the use of colorectal cancer-specific mortality should not affect our conclusion.

Despite these limitations, this study has several strengths. The data source of this study was based on a combination of the TCR and hospital clinical data. Therefore, the survival time and the cause of death could be accurately ascertained. In addition, CHM use patients' information, and comorbidities were obtained from the medical record database at a single regional hospital, thereby minimizing coding and misclassification errors.

## 5. Conclusions

This retrospective cohort study showed that integrated CHM and Western medicine was able to significantly improve survival in patients with colorectal cancer. In particular, Jia Wei Xiao Yao San, Zhi Bah Di Huang Wan, Ping Wei San, and Qui Pi Tang were significantly associated with better survival in patients with colorectal cancer. Findings from this study call for additional research on integrating TCM with Western medicine to improve cancer survival and reduce medical expenditures.

## Figures and Tables

**Table 1:** Comparison of age and sex distribution of the 1,209 patients with colorectal cancer identified from the Taiwan Cancer Registry and the 535 patients included in this study.

Variable	Source population (*N* = 1209)		Study sample (*N* = 535)		*P*
	No.	%	No.	%	
Age (years)					0.110
≤45	93	7.7	43	8.0	
46–65	442	36.5	192	35.9	
≥66	674	55.7	300	56.1	
Sex					0.969
Male	643	53.2	284	53.1	
Female	566	46.8	251	46.9	

**Table 2:** Characteristics of patients with colorectal cancer (*N* = 535).

Variable	CHM users (*N* = 147)		CHM nonusers (*N* = 388)		*P*
	No.	%	No.	%	
Age (years)					0.075
≤45	10	6.8	33	8.5	
46–65	64	43.5	128	33.0	
≥66	73	49.7	227	58.5	
Sex					0.851
Male	79	53.7	205	52.8	
Female	68	46.3	183	47.2	
Clinical stage					0.774
I	30	20.4	71	18.3	
II	22	15.0	67	17.3	
III	64	43.5	158	40.7	
IV	31	21.1	92	23.7	
Treatment					
Surgery	137	93.2	359	92.5	0.790
Radiotherapy	49	33.3	105	27.1	0.153
Chemotherapy	115	78.2	273	70.4	0.069
Distant metastases	31	21.1	92	23.7	0.520
Comorbidity score 1	18	12.2	22	5.7	0.010
Comorbidity score 2	8	5.4	20	5.2	0.894
Comorbidity score 6	9	6.1	18	4.6	0.659

CHM : Chinese herbal medicine. Comorbidity score 1: myocardial infarction, congestive heart failure, peripheral vascular disease, cerebrovascular disease, dementia, chronic pulmonary disease, rheumatologic disease, peptic ulcer disease, mild liver disease, and diabetes. Comorbidity score 2: diabetes with chronic complication, hemiplegia or paraplegia, renal disease, and malignancy (including leukemia and lymphoma). Comorbidity score 6: metastases solid tumor and acquired immune deficiency syndrome.

**Table 3:** Survival of patients with colorectal cancer (*N* = 535).

Variable	Unadjusted analysis			Adjusted analysis		
	HR	95% CI	*P*	Adjusted HR	95% CI	*P*
Chinese herbal medicine use						
Nonusers (<30d)	1.00			1.00		
Users (≥30 d)	0.62	0.44–0.88	0.007	0.54	0.38–0.77	0.001
Age (years)						
≤45	1.00			—	—	—
46–65	0.52	0.31–0.88	0.014	—	—	—
>65	0.95	0.59–1.52	0.820	—	—	—
Sex						
Female	1.00			—	—	—
Male	1.29	0.97–1.72	0.076	—	—	—
Clinical stage						
I	1.00			1.00		
II	3.25	1.58–6.70	0.001	2.98	1.44–6.18	0.003
III	3.30	1.69–6.42	<0.001	4.28	2.18–8.42	<0.001
IV	15.26	7.93–29.36	<0.001	17.49	8.90–34.38	<0.001
Surgery	0.18	0.12–0.26	<0.001	0.29	0.19–0.43	<0.001
Radiotherapy	0.82	0.60–1.12	0.215	—	—	—
Chemotherapy	0.76	0.56–1.02	0.069	0.47	0.34–0.65	<0.001
Distant metastases	5.69	4.27–7.57	<0.001	—	—	—
Comorbidity score 1	1.04	0.61–1.75	0.896	—	—	—
Comorbidity score 2	0.59	0.28–1.24	0.165	—	—	—
Comorbidity score 6	1.48	0.86–2.55	0.161	—	—	—

CI: confidence interval; HR: hazards ratio. Comorbidity score 1: myocardial infarction, congestive heart failure, peripheral vascular disease, cerebrovascular disease, dementia, chronic pulmonary disease, rheumatologic disease, peptic ulcer disease, mild liver disease, and diabetes. Comorbidity score 2: diabetes with chronic complication, hemiplegia or paraplegia, renal disease, and malignancy (including leukemia and lymphoma). Comorbidity score 6: metastases solid tumor and acquired immune deficiency syndrome.

**Table 4:** Top 10 commonly used Chinese herbal medicine formulae in patients with colorectal cancer (*N* = 147).

Formula	Ingredients	Function	No. of patients	%	Total dosage (g)
Liu Wei Di Huang Wan	*Radix Rehmanniae, Fructus corni officinalis, Radix Dioscoreae Oppositae, Paeonia suffruticosa Andr, Sclerotium Poriae Cocos, Alismatis Rhizoma*	Kidney yin deficient	40	27.2	14,328

Jia Wei Xiao Yao San	*Rhizoma Atractylodis Macrocephalae, Radix Glycyrrhizae Uralensis, Herba Menthae Haplocalycis, Radix Angelicae sinensis, Radix Paeoniae Lactiflorae, Rhizoma zingiberis officinalis, Radix Bupleuri, Cortex Moutan Radicis, Fructus Gardeniae Jasminoides*	Clears liver Qi stagnation and heat	74	50.3	41,437

Ban Xia Xie Xin Tang	*Rhizoma Pinelliae Ternatae, Radix Scutellariae Baicalensis, Radix Ginseng, Radix Glycyrrhizae Uralensis, Rhizoma zingiberis officinalis, Rhizoma Coptidis (Huang Lian), Fructus Zizyphi Jujubae*	Harmonize the stomach and descend the rebellious Qi	45	30.6	17,617

Ping Wei San	*Atractylodes Rhizome, Magnolia Bark, Dried Tangerine Peel, Licorice Root, Fructus Zizyphi Jujubae*	Dries dampness and harmonizes spleen-stomach relationship	70	47.6	22,445

Zhi Gan Cao Tang	*Radix Glycyrrhizae Uralensis, Rhizoma zingiberis officinalis, Ramulus Cinnamomi Cassiae, Radix Ginseng, Colla Corrii Asini, Tuber Ophiopogonis Japonici, semen cannabis sativae, Fructus Zizyphi Jujubae*	Anemic and yang deficient syndrome	31	21.1	12,498

Zhi Bai Di Huang Wan	*Radix Rehmanniae, Fructus corni officinalis, Radix Dioscoreae Oppositae, Paeonia suffruticosa Andr., Sclerotium Poriae Cocos, Alismatis Rhizoma, Anemarrhena Rhizome, Cortex Phellodendri*	Yin deficient and heat is high	34	23.1	13,274

Li Zhong Tang	*Rhizoma Atractylodis Macrocephalae, Radix Ginseng, Radix Glycyrrhizae Uralensis, Rhizoma zingiberis officinalis*	Spleen deficient	56	38.1	22,413

Du Huo Ji Sheng Tang	*Radix Angelicae Pubescentis, Ramulus Sangjisheng, Cortex Eucommiae Ulmoidis, Herba cum Radice Asari, Radix Gentianae macrophylliae, Sclerotium Poriae Cocos, Ramulus Cinnamomi Cassiae, Radix Saposhnikoviae divaricatae, Radix Ligustici Chuanxiong, Radix Ginseng, Radix Glycyrrhizae Uralensis, Radix Angelicae sinensis, Radix Paeoniae Lactiflorae, Radix Rehmanniae*	Gets rid of wind dampness, pain due to Qi and blood stasis, tonic for Qi and blood	15	10.2	13,182

Qui Pi Tang	*Radix Ginseng, Sclerotium Poriae Cocos, Rhizoma Atractylodis Macrocephalae, Radix astragali, Semen Zizyphi Spinosae, Arillus Euphoriae Longanae, Radix Plygalae Tenuifoliae, Radix Glycyrrhizae Uralensis, Fructus Zizyphi Jujubae, Radix Aucklandiae, Rhizoma zingiberis officinalis*	Spleen deficient, blood deficient	46	31.3	14,676

Huo Xiang Zhen Qi San	*Pericarpium Arecae Catechu, Radix Angelicae Dahuricae, Folium Perillae Frutescentis, Rhizoma Pinelliae Ternatae, Pericarpium Citri Reticulatae, Cortex Magnoliae Officinalis, Radix Platycodi grandiflora, Herba Agastaches seu Pogostemi, Radix Glycyrrhizae Uralensis*	Relieves dampness	54	36.7	14,821

**Table 5:** Effects of 10 most commonly used Chinese herbal medicine formulae on the survival of patients with colorectal cancer (*N* = 535).

Formula (n)	Unadjusted analysis			Adjusted analysis		
	HR	95% CI	*P*	Adjusted HR	95% CI	*P*
Jia Wei Xiao Yao San						
Not used(476)	1.00			1.00		
Low dose (29)	0.77	0.40–1.51	0.446	0.40	0.20–0.79	0.009
High dose (30)	0.34	0.14–0.82	0.017	0.38	0.16–0.93	0.035
Liu wei Di huang Wan						
Not used (503)	1.00			1.00		
Low dose (16)	0.44	0.14–1.37	0.158	0.64	0.20–2.01	0.443
High dose (16)	0.12	0.02–0.89	0.038	0.18	0.02–1.28	0.086
Zhi Bai Di Huang Wan						
Not used (507)	1.00			1.00		
Low dose (15)	0.74	0.30–1.79	0.497	0.46	0.20–1.13	0.091
High dose (13)	0.15	0.02–1.09	0.061	0.15	0.02–1.05	0.056
Ban Xia Xie Xin Tang						
Not used (500)	1.00			1.00		
Low dose (19)	0.72	0.32–1.62	0.424	0.49	0.21–1.14	0.096
High dose (16)	0.41	0.13–1.28	0.126	0.32	0.10–1.00	0.049
Ping Wei San						
Not used (475)	1.00			1.00		
Low dose (41)	0.83	0.47–1.45	0.505	0.56	0.32–1.01	0.054
High dose (19)	0.45	0.17–1.20	0.110	0.31	0.11–0.84	0.022
Zhi Gan Cao Tang						
Not used (508)	1.00			1.00		
Low dose (13)	0.18	0.03–1.28	0.086	0.19	0.03–1.36	0.099
High dose (14)	0.45	0.14–1.40	0.165	0.48	0.15–1.50	0.204
Li Zhong Tang						
Not used (491)	1.00			1.00		
Low dose (20)	0.63	0.26–1.52	0.302	0.47	0.19–1.16	0.101
High dose (24)	0.42	0.17–1.03	0.057	0.50	0.20–1.22	0.130
Du Huo Ji Sheng Tang						
Not used (527)	1.00			1.00		
Low dose (2)	NC	NC	NC	NC	NC	NC
High dose (6)	0.31	0.04–2.20	0.240	0.47	0.07–3.38	0.453
Qui Pi Tang						
Not used (500)	1.00			1.00		
Low dose (17)	1.21	0.54–2.73	0.650	1.27	0.56–2.87	0.571
High dose (18)	0.78	0.34–1.75	0.544	0.26	0.11–0.59	0.001
Huo Xiang Zhen Qi San						
Not used (494)	1.00			1.00		
Low dose (22)	1.31	0.67–2.56	0.428	1.08	0.55–2.14	0.819
High dose (19)	0.48	0.18–1.29	0.145	0.66	0.24–1.78	0.407

CI: confidence interval; HR: hazards ratio; NC: not calculable.

**Table 6:** Daily dosage and duration of Chinese herbal medicine administration.

Formula	Dose	Daily dosage^a^ (g)	Duration^b^ (days)
Jia Wei Xiao Yao San	High	4.59	63
	Low	4.09	25
Zhi Bai Di Huang Wan	High	4.50	57

Ping Wei San	High	2.92	58
Qui Pi Tang	High	4.68	33

Patients taking dosage above and below the median were classified as high- or low-dose group, respectively. ^a^The daily dosage of the Chinese herbal medicine administration was calculated as follows: total dosage/total days. ^b^The duration of the Chinese herbal medicine administration required for the high- and low-dose groups was calculated as follows: (median dose/daily dosage of the high-dose group) and (total dosage/daily dosage of the low-dose group).

## Data Availability

The data used to support the findings of this study are available from the corresponding author upon request.

## Abbreviations

AJCC: American Joint Committee on Cancer
CHM: Chinese herbal medicine
CI: Confidence interval
ICD-9-CM: International Classification of Disease, Clinical modification, 9th edition
HR: Hazard ratio
NHIRD: National Health Insurance Research Database
SD: Standard deviation
TCM: Traditional Chinese Medicine
TCR: Taiwan Cancer Registry.
